# Selection and testing of reference genes for accurate RT-qPCR in rice seedlings under iron toxicity

**DOI:** 10.1371/journal.pone.0193418

**Published:** 2018-03-01

**Authors:** Fabiane Igansi de Castro dos Santos, Naciele Marini, Railson Schreinert dos Santos, Bianca Silva Fernandes Hoffman, Marcio Alves-Ferreira, Antonio Costa de Oliveira

**Affiliations:** 1 Plant Genomics and Breeding Center, Universidade Federal de Pelotas, Capão do Leão, Brazil; 2 Embrapa Uva e Vinho, Bento Gonçalves, Brazil; 3 Universidade Federal do Rio de Janeiro, Rio de Janeiro, Brazil; Louisiana State University, UNITED STATES

## Abstract

Reverse Transcription quantitative PCR (RT-qPCR) is a technique for gene expression profiling with high sensibility and reproducibility. However, to obtain accurate results, it depends on data normalization by using endogenous reference genes whose expression is constitutive or invariable. Although the technique is widely used in plant stress analyzes, the stability of reference genes for iron toxicity in rice (*Oryza sativa* L.) has not been thoroughly investigated. Here, we tested a set of candidate reference genes for use in rice under this stressful condition. The test was performed using four distinct methods: NormFinder, BestKeeper, geNorm and the comparative ΔCt. To achieve reproducible and reliable results, Minimum Information for Publication of Quantitative Real-Time PCR Experiments (MIQE) guidelines were followed. Valid reference genes were found for shoot (*P2*, *OsGAPDH* and *OsNABP*), root (*OsEF-1a*, *P8* and *OsGAPDH*) and root+shoot (*OsNABP*, *OsGAPDH* and *P8*) enabling us to perform further reliable studies for iron toxicity in both indica and japonica subspecies. The importance of the study of other than the traditional endogenous genes for use as normalizers is also shown here.

## Introduction

The reverse transcription quantitative PCR (RT-qPCR) has revolutionized the field of gene expression analysis. Being one of the most sensitive, precise and reproducible techniques to quantify specific RNAs, it is now employed in diverse fields and became the most common method for validating microarray and RNAseq data [1–3]. Although it is an extremely powerful technique, RT-qPCR suffers from certain pitfalls, and the normalization with a housekeeping gene, which expression remains constant between cells of different tissues and under different experimental conditions, is an important step of this analysis [4].

Some genes involved in basic cellular processes are commonly used as endogenous controls for gene expression analyses as they are supposed to have a uniform expression. However, since some studies began to demonstrate that the transcript levels of these genes can also vary considerably, the need to identify genes with higher expression stability became more evident [5–7]. Different statistical algorithms and methods such as geNORM, NormFinder, BestKeeper and comparative ΔCt (cycle threshold) have been developed to evaluate the best suited housekeeping genes for normalization of RT-qPCR data in a given set of samples [8–11].

Asian rice (*Oryza sativa* L.) is one of the world’s main staple foods and significant improvement of grain productivity of this crop is essential to meet the growing demand for food. Climate change will impact the extent and productivity of both irrigated and rainfed agriculture across the globe, intensifying the damage caused by both biotic and abiotic stresses. However, the effects of abiotic stresses are expected to be greater [12].

Among the most important abiotic stresses in rice is iron toxicity, a root related stress, that is usually associated with flooded soils and consequently with lowland production systems. In the absence of oxygen high concentrations of reduced iron (Fe^2+^) become available in soil solution and the excessive uptake of this element can damage rice plants in several ways [13].

Scarce reports related to molecular responses to the excess of this element are available, while its deficiency is much more commonly studied [14,15]. It is known that measuring transcriptional expression responses to this stress can aid plant breeding [16,17]. However, in order to achieve deeper understanding on this phenomenon, we need to define which are the best housekeeping genes for this condition.

In this study we have investigated the transcriptional stability of housekeeping genes frequently used in rice (*OsGAPDH*, *OsNABP*, *OsEF-1a*) in distinct plant organs and genotypes, comparing their expression in conditions of iron stress against control conditions. Moreover, nine novel candidate genes identified from microarray databases were proposed and these also had the stability evaluated according to four different methods.

## Materials and methods

### Plant materials and iron treatment

Indica cultivars with different levels of sensitivity to iron (BR IRGA 409—sensitive and Epagri 108—tolerant) and a control/model japonica cultivar (Nipponbare) were used in this study. The seeds were disinfected in HCl 1% and distributed in acrylic boxes with moistened germination paper. The boxes were placed in germination chambers (25°C; 6 hours light/8 hours of dark; 100% RH) for seven days. Therefore, seedlings were then transferred to vessels (2 L) containing nutrient solution [18]: 91.4 g L^-1^ NH_4_NO_3_, 40.3 g L^-1^ NaH_2_PO_4_.2H_2_O, 71.4 g L^-1^ K_2_SO_4_, 88.6 g L^-1^ CaCl_2_, 324.0 g L^-1^ MgSO_4_.7H_2_O, 1.5 g L^-1^ MnCl_2_.4H_2_O, 0.074 g L^-1^ (NH_4_)^6^MO_7_O_24_.4H_2_O, 0.934 g L^-1^ H_3_BO_3_, 0.035 g L^-1^ ZnSO_4_.7H_2_O, 0.031 g L^-1^ CuSO_4_.5H_2_O, 7.7 g L^-1^ FeCl_3_.6H_2_O and 11.9 g L^-1^ citric acid monohydrate, pH 5.0 ± 0.1. The pH was adjusted and to keep it constant, 2-(N-morpholino) ethanesulfonic acid buffer (MES) was used. During the period in which the plants were kept in nutrient solution the photoperiod was 16 hours of light and 8 hours of dark.

The solution was changed after 7 days and in 14^th^-day seedlings were subjected to iron treatment (500 mg L^-1^ FeSO_4_.7H_2_O at pH 4.0 ± 0.1), according to a previously described protocol [19]. The experiment consisted of three replicates of each treatment (iron and control) for each cultivar (BR IRGA 409, Epagri 108 and Nipponbare) in a completely randomized design, where the experimental units were daily randomly rearranged. Shoots and roots were collected separately at 12, 24 and 36 hours after the application of stress. Samples were immediately frozen in liquid nitrogen and stored at -80°C for RNA extraction.

### Total RNA extraction and cDNA synthesis

Total RNA was extracted in biological triplicates from 0.1 g of shoot or root tissues from rice seedlings following the protocol described by Trizol^™^ reagent (Invitrogen^™^, Carlsbad, CA, USA). The quantity of the RNA was assessed spectroscopically and the quality was assessed by agarose gel electrophoresis. Genomic DNA was eliminated by DNAse I (Invitrogen^™^, Carlsbad, CA, USA) treatment, as recommended by the manufacturer. Each sample was reverse-transcribed into cDNA using the commercial kit SuperScript^®^ III First-Strand System for RT-qPCR (Invitrogen^™^, Carlsbad, CA, USA), according to the manufacturer recommendations.

### Primer selection, design, and RT-qPCR conditions

The RefGenes tool from Genevestigator was used in order to identify new candidate reference genes [20]. Due to the absence of experiments with stress by iron excess, conditions of iron deficiency were used in the search of these genes. Genes with low variation in Genevestigator and confirmed by our group as less variable were chosen [16].

The RT-qPCR followed the guidelines of the Minimum Information for Publication of Quantitative Real-Time PCR Experiments (MIQE) [21]. The design of primers for RT-qPCR analysis was performed using the Applied Biosystems Primer Express^®^ program according to the following parameters: annealing temperature of 60–65°C, GC content of 40–60% and amplicon size of 50–150 bps. All primer sequences and relevant information are presented in Table 1. Dissociation curves were evaluated and only primers giving single peaks were used.

**Table 1:** Genome and amplification information on the candidate reference genes 1–10**.

Gene description / acronym*	ID*	Primer Sequence (5’ to 3’)		Amplicon
		Forward	Reverse	Size (bp)
*Genevestigator Gene 1* (*P1*)	Os01t0220700-00	TTTGTGTTGCGAATGGTGCT	TGCTGTCAGCGCAAAGAATG	51
*Genevestigator Gene 2* (*P2*)	Os01t0252200-01	ATCGGCGTCTGCCAACACT	ATCCCCAAACTTGCACGTCC	51
*Genevestigator Gene 3* (*P3*)	Os01t0654300-01	ATCCTGGGACCATAGAGCCG	CAGCCGGATGCTGATTCTGT	51
*Genevestigator Gene 4* (*P4*)	Os01t0759500-01	TGCATCGCCTCCGTTACGAC	CCCAGACGAAGAACCGGAAG	51
*Genevestigator Gene 5* (*P5*)	Os05t0352700-00	CGAGTGATTGGAGCATGGCA	TTGCGCTATGGACCCAGCT	51
*Genevestigator Gene 6* (*P6*)	Os07t0644200-01	GAAATTCATTGCCGAAAGCC	CACATAACGCAGCTCCTCATG	51
*Genevestigator Gene 8* (*P8*)	Os09t0560400-01	GGATCCCAACAAGCCCTCCT	CAGCCGATTCATCATTGCATACT	51
*Genevestigator Gene 9* (*P9*)	Os10t0378400-01	CCCATCTGGTTCGAATCGAA	TTTTAACCGACCAACCGATTG	51
*Genevestigator Gene 10* (*P10*)	Os12t01119600-00	AGGATAAACCCATCGAGGCC	GTGAAGCACAAAGGATGCCA	51
*Glyceraldehyde 3-Phosphate Dehydrogenase* (*OsGAPDH*)	Os04t0606400-02	AAGCCAGCATCCTATGATCAGATT	CGTAACCCAGAATACCCTTGAGTTT	79
*Nucleic Acid Binding Protein* (*OsNABP)*	Os06g0215200	GGAATGTGGACGGTGACACT	TCAAAATAGAGTCCAGTAGATTTGTCA	100
*Rice Elongation Factor 1 alpha* (*OsEF-1a*)	Os03t0177500-01	TGGTATGGTGGTGACCTTTG	GTACCCACGCTTCAGATCCT	151

* GenBank database or compositae genome project database (http://cgpdb.ucdavis.edu/);

**Genevestigator Gene 7 (P7) was discarded due to unreliable peaks.

Each reaction was performed in triplicate in an ABI 7500 Fast Real-Time PCR System using SYBR R Green I (Life Technologies^®^, Carlsbad, CA, USA). Each PCR reaction mix consisted of 2 μL of SYBR Green (1:10000), 0.4 μL of forward and reverse oligos (10 mM), 2 μL of PCR buffer (10x), 0.05 μL of dNTPs (10 mM), 1.2 μL of MgCl_2_ (50 mM) and 0.05 μL of Platinum R Taq DNA Polymerase (Life Technologies^®^, Carlsbad, CA, USA; 2 U/rxn) in a total volume of 10 μL. Finally, 10 μL of 1:50 diluted template cDNA was added, resulting in a total volume of 20 μL per PCR reaction. PCR cycling was performed as follows: 5 min at 94°C followed by 40 rounds of 15 s at 94°C, 10 s at 60°C, 15 s at 72°C, and finally 1 round of 35 s at 60°C. Melting curve cycling consisted of: 15 s at 95°C, 1 min at 60°C, 30 s at 95°C, and 15 s at 60°C.

### Data analysis

Real-time PCR Miner [22] was used to evaluate primer efficiency. The efficiency (E) of each primer pair was greater than 90% in all experimental sets, indicating that the amount of PCR product nearly doubled after each cycle. The Ct values were submitted, trough RefFinder [23] (http://fulxie.0fees.us), to four different methods that rank the best constitutive genes, geNorm [9], NormFinder [10], BestKeeper [11] and the comparative ΔCt method [24]. The softwares are based on different algorithms using information as Ct analysis of the genes in different tissues and groups of three biological replicates and initiator efficiency in each biological sample. This analysis identified genes that show the smallest variation in the number of transcripts in each tissue. The relative expression data were calculated according to the 2^-ΔΔCt^ method [25].

## Results

In this study, we tested three traditional reference genes used in many previous studies (*OsGAPDH*, *OsNABP* and *OsEF-1a*) and nine novel candidate reference genes selected from the Genevestigator database (Table 1). The transcriptional stability of the candidates was evaluated using four methods: geNorm, NormFinder, BestKeeper and the comparative ΔCt method.

### Amplification efficiency of the selected primers

The specificity of the primers was confirmed by the presence of single peaks in the dissociation curves of every primer. The efficiency of amplification for the primers in RT-qPCR reactions ranged from 1.8 to 2.0 for all tissues, genotypes and experimental conditions (Table 2).

**Table 2:** Efficiency of primer pairs used for RT-qPCR amplification in each experiment.

Primers	Shoots	Roots
*P1*	1.91	1.94
*P2*	1.90	1.87
*P3*	1.90	1.88
*P4*	1.89	1.89
*P5*	1.88	1.89
*P6*	1.88	1.88
*P8*	1.88	1.9
*P9*	1.93	1.88
*P10*	1.87	1.88
*EF1*	1.89	1.87
*OsGAPDH*	1.84	1.85
*OsNABP*	1.91	1.88

### Expression levels of candidate reference genes

**Fig 1:**
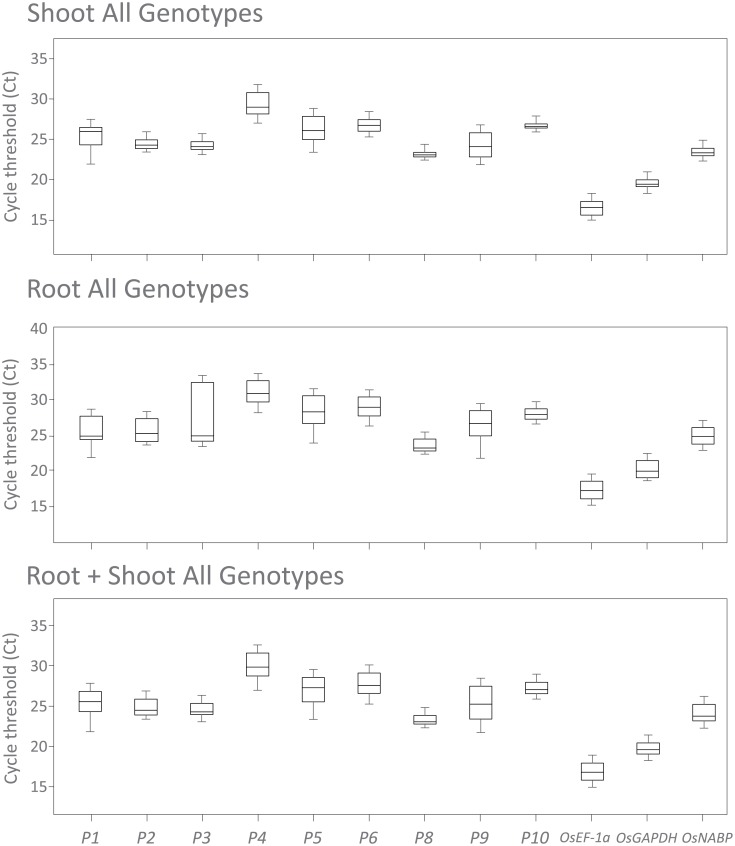
Gene expression levels of the candidate reference genes in rice. The genes, *OsEF-1a* (Os03t0177500-01), *P1* (Os01t0220700-00), *P2* (Os01t0252200-01), *P3* (Os01t0654300-01), *P4* (Os01t0759500-01), *P5* (Os05t0352700-00), P6 (Os07t0644200-01), *P8* (Os09t0560400-01), *P9* (Os10t0378400-01), P10 (Os12t01119600-00), *GAPDH* (Os04t0606400-02), and *OsNABP* (Os06g0215200) were evaluated in shoots and roots of one japonica (Nipponbare) and two contrasting indica (Epagri 108 and IRGA 409) cultivars subjected to iron treatment (500 mg L^-1^ FeSO_4_.7H_2_O at pH 4.0 ± 0.1), for 0, 12, 24 and 36 hours. Horizontal bars represent Ct median and whiskers represent highest and lowest values.

Transcriptional stability is dependent on the Ct values of the candidate genes throughout the experimental conditions. For *P3*, Ct values were highly variable, ranging from 23.11 to 36.13 considering the different tissues, genotypes and experimental conditions (Fig 1). The most variable levels of transcription for *P3* were observed in roots subjected to iron stress (Fig 1). Transcript accumulation for *OsEF-1a*, a commonly employed normalizer gene in RT-qPCR experiments, was highly stable in roots, but not in shoots of rice under iron excess (Fig 1). On the other hand, the smaller variations of Ct values in the tested experimental conditions were observed for *OsGAPDH* and *OsNABP* (Fig 1).

### Transcription stability analyses

#### geNorm

**Fig 2:**
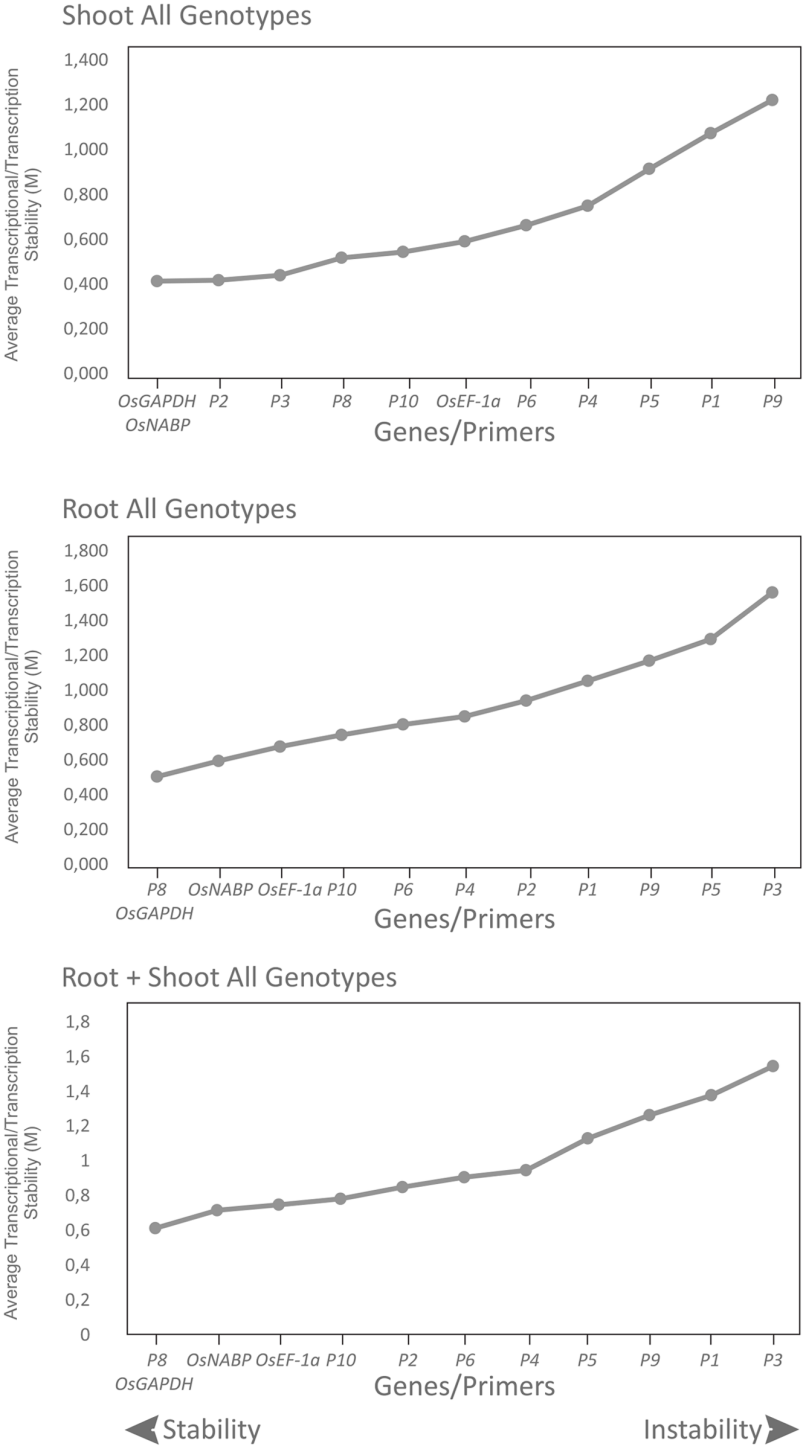
Transcriptional stability of the candidate reference genes investigated by geNorm. The candidate reference genes, *OsEF-1a* (Os03t0177500-01), *P1* (Os01t0220700-00), *P2* (Os01t0252200-01), *P3* (Os01t0654300-01), *P4* (Os01t0759500-01), *P5* (Os05t0352700-00), *P6* (Os07t0644200-01), *P8* (Os09t0560400-01), *P9* (Os10t0378400-01), *P10* (Os12t01119600-00), *GAPDH* (Os04t0606400-02), and *OsNABP* (Os06g0215200) were evaluated in shoots and roots of one japonica (Nipponbare) and two contrasting indica (Epagri 108 and IRGA 409) cultivars subjected to iron treatment (500 mg L^-1^ FeSO_4_.7H_2_O at pH 4.0 ± 0.1), for 0, 12, 24 and 36 hours.

The results of M value analyses demonstrated that *P3* is not a suitable reference gene for roots, with an M value of 1.561, nor for root + shoot, where its M value was 1.545 (Table 3). The recommended normalizers by geNorm can be seen in Table 3 and Fig 2. Based on these results it is possible to notice that the *OsGAPDH* gene is suitable for shoot, root as well as for shoot + root. Two other suitable genes for qPCR analysis were *OsNABP* and *P8*.

**Table 3:** Ranking of the 12 candidate reference genes according to their transcription stability.

Software	Comprehensive		NormFinder		geNorm		BestKeeper		ΔCt	
	Ranking	Geomean of ranking values	Ranking	Stability value	Ranking	Stability value	Ranking	coeff. of corr. [r]	Ranking	Average of STDEV
**Shoot All Genotypes**	*P2*	1.97	*P2*	0.312	*GAPDH* | *OsNABP*	0.413	*OsEF-1a*	0.855	*P2*	0.89
	*OsGAPDH*	2.45	*OsNABP*	0.411	*P2*	0.417	*P1*	0.484	*P3*	0.94
	*OsNABP*	2.63	*OsGAPDH*	0.422	*P3*	0.439	*P2*	0.911	*OsGAPDH*	0.95
	*P3*	3.13	*P3*	0.452	*P8*	0.517	*P3*	0.862	*OsNABP*	0.96
	*P10*	4.28	*P6*	0.560	*P10*	0.543	*P4*	0.959	*P8*	1.02
	*P8*	4.33	*OsEF-1a*	0.585	*OsEF-1a*	0.590	*P5*	0.612	*OsEF-1a*	1.03
	*OsEF-1a*	6.48	*P8*	0.623	*P6*	0.662	*P6*	0.862	*P10*	1.07
	*P6*	7.11	*P10*	0.715	*P4*	0.749	*P8*	0.731	*P6*	1.07
	*P4*	9.00	*P4*	0.811	*P5*	0.914	*P9*	0.865	*P4*	1.22
	*P5*	10.24	*P5*	1.445	*P1*	1.073	*P10*	0.640	*P5*	1.68
	*P1*	10.74	*P1*	1.669	*P9*	1.221	*OsGAPDH*	0.880	*P1*	1.86
	*P9*	12.00	*P9*	1.791			*OsNABP*	0.866	*P9*	1.96
**Root All Genotypes**	*OsEF-1a*	2.21	*OsEF-1a*	0.499	*P8* | *OsGAPDH*	0.504	*OsEF-1a*	0.956	*OsEF-1a*	1.21
	*P8*	2.63	*P6*	0.664	*OsNABP*	0.594	*P1*	0.793	*OsNABP*	1.24
	*OsGAPDH*	2.63	*OsNABP*	0.668	*OsEF-1a*	0.676	*P2*	0.946	*OsGAPDH*	1.25
	*OsNABP*	2.71	*OsGAPDH*	0.691	*P10*	0.743	*P3*	0.905	*P8*	1.29
	*P10*	4.09	*P4*	0.700	*P6*	0.803	*P4*	0.929	*P6*	1.29
	*P6*	4.16	*P8*	0.798	*P4*	0.849	*P5*	0.819	*P4*	1.33
	*P4*	6.19	*P2*	0.830	*P2*	0.940	*P6*	0.931	*P10*	1.44
	*P2*	7.97	*P10*	1.033	*P1*	1.053	*P8*	0.923	*P2*	1.44
	*P1*	9.21	*P9*	1.242	*P9*	1.169	*P9*	0.935	*P9*	1.71
	*P9*	9.72	*P1*	1.344	*P5*	1.293	*P10*	0.856	*P1*	1.72
	*P5*	10.74	*P5*	1.536	*P3*	1.561	*OsGAPDH*	0.931	*P5*	1.92
	*P3*	12.00	*P3*	2.750			*OsNABP*	0.939	*P3*	2.90
**Root + Shoot All Genotypes**	*OsNABP*	1.86	*OsNABP*	0.539	*P8* | *OsGAPDH*	0.613	*OsEF-1a*	0.926	*OsNABP*	1.21
	*OsGAPDH*	2.34	*P2*	0.612	*OsNABP*	0.716	*P1*	0.605	*OsEF-1a*	1.25
	*P8*	2.55	*OsEF-1a*	0.614	*OsEF-1a*	0.748	*P2*	0.944	*OsGAPDH*	1.26
	*OsEF-1a*	3.31	*P6*	0.657	*P10*	0.782	*P3*	0.892	*P2*	1.29
	*P2*	4.28	*OsGAPDH*	0.679	*P2*	0.849	*P4*	0.929	*P6*	1.30
	*P6*	5.38	*P4*	0.757	*P6*	0.906	*P5*	0.810	*P8*	1.34
	*P10*	5.57	*P8*	0.867	*P4*	0.946	*P6*	0.913	*P4*	1.35
	*P4*	7.42	*P10*	0.876	*P5*	1.129	*P8*	0.879	*P10*	1.37
	*P5*	9.24	*P5*	1.528	*P9*	1.263	*P9*	0.897	*P5*	1.88
	*P1*	10.16	*P9*	1.558	*P1*	1.377	*P10*	0.854	*P9*	1.90
	*P9*	10.24	*P1*	1.715	*P3*	1.545	*OsGAPDH*	0.923	*P1*	1.99
	*P3*	12.00	*P3*	2.162			*OsNABP*	0.936	*P3*	2.38

#### NormFinder

The ANOVA based algorithm of NormFinder generated a similar rank of recommended reference genes for transcriptional analyses using distinct tissues (Table 3). The classification of the most stably transcribed genes in response to iron was more similar for geNorm and NormFinder than for BestKeeper (Table 3). The results of NormFinder demonstrate that the most stable genes differ when different tissues are compared, evidencing the importance of the definition of endogenous references that are specific to each tissue. In the case of shoot the best gene was *P2*, with stability value of 0.312, while in root it was *OsEF-1a* (0.499) and in root + shoot *OsNABP* (0.539).

#### BestKeeper

BestKeeper indicates distinct sets of genes as the most suitable references for each tissue. The transcription of the candidates was highly responsive to the tissue, still *OsNABP* seems to be the best gene when comparing different tissues trough this software (Table 3, S1 Table). When considering root + shoot the CV values were also high for *P3* (9.55), *P9* (8.62) and *P5* (7.09). On the other hand, considering only roots, the worst CVs were *P3* (13.75), *P9* (8.27) and *OsEF-1a* (7.95). The most stable CV values were found in shoot tissues, where the least stable genes were *P9* (7.59), *P1* (5.59) and *OsEF-1a* (5.50). General results by BestKeeper indicate *OsNABP* and *P2* as the most stable genes (Table 3).

#### ΔCt method

The results of the ΔCt method are reported in Table 3. *P2* was the most stable gene for shoot (with an average standard deviation of 0.89), *OsEF-1a* for root (1.21) and *OsNABP* for root + shoot (1.21). However, if we considered the five most stable genes in each condition, the genes *OsGAPDH* and *OsNABP* appear recurrently, showing high stability in different organs.

## Discussion

In plant stress research, studies on gene expression patterns are important for understanding the biological processes involved in stress responses. Since RT-qPCR has become the primary quantitative method for accurate expression profiling of target genes, the requirement of a normalization method against reference genes is essential to achieve reliable results. According to MIQE guidelines [21], the use of reference genes as internal controls is the best normalization strategy [26]. Since there are no universal reference genes and the number of genes to use can vary, these parameters need to be experimentally determined [21,27]. Although many reference genes have been determined for evaluation of different plant tissues, genotypes and stresses [27–29], to the best of our knowledge, no study assessed reference genes for evaluation of transcriptional responses to iron excess in rice. Here, the transcriptional stability of 12 candidate genes, consisting of three commonly used and nine novel proposed references was investigated. The rice transcriptional profiling was assessed by the comparative analyses of the results generated using three software packages with data from three genotypes and two different tissues. The transcriptional stability of different reference genes has been investigated in plants [30–34]. These results suggest that transcriptional stability is dependent on tissue, genotype and the experimental conditions to which it has been subjected, and that the use of multiple reference genes is recommended for better normalization. Although studies of gene expression through RT-qPCR have been made with rice under stress by iron [13], this represents the first effort to investigate the transcriptional behavior of reference genes in rice under iron excess, an important condition of abiotic stress that is still neglected by studies in the field of molecular biology.

Distinct tools are used to investigate the transcriptional stability of candidate reference genes [9–11]. Here we employ four methods (geNorm, Norm-Finder, BestKeeper and the comparative ΔCt method), to study the transcriptional profile of different genes in two tissues of three genotypes under iron stress. The comparative analysis of the transcription stability results by the different packages demonstrated some differences in candidate gene ranking (Table 3), something that can also be observed in other studies [30,35]. These results combined in the online package RefFinder [23] (http://fulxie.0fees.us) offer an overall comprehensive analysis based on the four methods shown here (Table 4). Although the ranking presents some variations, the genes considered to be the most stable in each tissue were similar. Large discrepancies were not noticed when comparing packages.

**Table 4:** Recommended comprehensive ranking.

	Genes/Primers											
	1°	2°	3°	4°	5°	6°	7°	8°	9°	10°	11°	12°
**Root + Shoot All Genotypes**	***OsNABP***	***GAPDH***	***P8***	*OsEF-1a*	*P2*	*P6*	*P10*	*P4*	*P5*	*P1*	*P9*	*P3*
**Shoot All Genotypes**	***P2***	***GAPDH***	***OsNABP***	*P3*	*P10*	*P8*	*OsEF-1a*	*P6*	*P4*	*P5*	*P1*	*P9*
**Root All Genotypes**	***OsEF-1a***	***P8***	***GAPDH***	*OsNABP*	*P10*	*P6*	*P4*	*P2*	*P1*	*P9*	*P5*	*P3*

Iron excess can cause deep metabolic alterations in rice tissues, changing the transcriptional expression of different genes [16,17]. Thus, the transcriptional profile of the candidate reference genes was investigated in distinct plant tissues and genotypes providing the identification of valid reference genes for shoot (*P2*, *OsGAPDH* and *OsNABP*), Root (*OsEF-1a*, *P8* and *GAPDH*) and Root+Shoot (*OsNABP*, *OsGAPDH* and *P8*) enabling us to perform further reliable studies for iron toxicity in both indica and japonica subspecies.

The gene *OsGAPDH* codes for glyceraldehyde 3-phosphate dehydrogenase, an enzyme that catalyzes the sixth step of glycolysis, the first stage of respiration, and thus serves to break down glucose for energy and carbon molecules. Although iron is an essential nutrient for plants also being involved in respiration, accepting and donating electrons and playing important roles in the electron-transport chains, it seems not to affect the transcriptional activity of *OsGAPDH*, an stability that can also be noticed for other different stresses [27,28,36].

The *OsNABP* (Os06g0215200), a zinc finger protein capable of binding to nucleic acids, showed to be stable in both organs (roots and shoots) and, along with the *OsGAPDH*, is always among the four best normalizers in the different organs analyzed here.

In shoots, the gene *P2* (Os01t0252200-01, also known as *OsC3H3*) is highly stable, constituting a new alternative for the normalization of RT-qPCR analyzes. This gene is also annotated as a zinc finger protein with a domain of the CCCH type. Genes of this family encode proteins containing motifs with three cysteines and one histidine residues and are known to play important roles in RNA processing. Few plant CCCH proteins have been studied functionally and *P2*/*OsC3H3* is one of these [37].

In roots, *OsEF-1a* presented the highest stable levels of expression, similar to results obtained for sugarcane (*Saccharum* spp. hybrids) under salinity and drought stresses [28]. The eukaryotic elongation factor 1 complex has many roles in different organisms [38], however, what stands out is that its stability of expression causes it to be commonly used as an endogenous reference [2,28,39].

Another novel reference gene here reported to be stable under iron stress conditions, the *P8* (Os09t0560400-01), is a hypothetical conserved gene with unknown roles. Although the evaluation of reference genes lingers on well-known options, this result shows that the evaluation of transcripts of unfamiliar loci may bring information useful for the normalization of RT-qPCR analyzes.

## Conclusions

The best genes for normalization of RT-qPCR in rice under iron excess are *GAPDH*, *OsNABP*, *P2* and *P8*, where *P2* and *P8* represent a novel, viable, alternative for data normalization in rice. The ranking results were similar when comparing the different investigated methods. Our results reinforce the importance of the determination of transcription stability of different candidate genes considering each experimental condition of interest.

## Supporting information

S1 Table Descriptive statistic analyses by BestKeeper. Transcriptional regulation of twelve candidate reference genes tested in shoots and roots of one japonica (Nipponbare) and two contrasting indica (Epagri 108 and IRGA 409) cultivars subjected to iron treatment (500 mg L^-1^ FeSO_4_.7H_2_O at pH 4.0 ± 0.1), for 0, 12, 24 and 36 hours, using the software BestKeeper. Absent data (-) corresponds to unviable calculations due to differentially regulated transcription under the given experimental conditions.
(XLSX)
